# The blood pressure and use of tourniquet are related to local recurrence after intralesional curettage of primary benign bone tumors: a retrospective and hypothesis-generating study

**DOI:** 10.1186/s12891-022-05157-4

**Published:** 2022-03-03

**Authors:** Lenian Zhou, Shanyi Lin, Hongyi Zhu, Yang Dong, Qingcheng Yang, Ting Yuan

**Affiliations:** Department of Orthopaedics, Shanghai Jiao Tong University Affiliated Sixth People’s Hospital, 600 Yishan Road, Shanghai, 200233 China

**Keywords:** Blood pressure, Bone tumors, Curettage, Local recurrence, Tourniquet

## Abstract

**Aims:**

Intralesional curettage is a commonly used treatment for primary bone tumors. However, local recurrence of tumors after curettage remains a major challenge.

**Questions:**

(1) Is blood pressure related to local recurrence after intralesional curettage for benign or intermediate bone tumors? (2) What’s the impact of tourniquet usage on the risk of recurrence from high blood pressure?

**Methods:**

This retrospective study evaluated patients receiving intralesional curettage for primary bone tumors from January 2011 to January 2015. A total of 411 patients with a minimum five-year follow-up were included for analysis. Demographic and disease-related variables were first assessed in univariable analyses for local recurrence risk. When a yielded *p*-value was < 0.2, variables were included in multivariable analyses to identify independent risk factors for local recurrence. Patients were then stratified by tourniquet usage (use/non-use), and risk from high blood pressure was evaluated in both subgroups.

**Results:**

At an average follow-up of 6.8 ± 1.0 years, 63 of 411 patients (15.3%) experienced local recurrence. In multivariable analyses, local recurrence was associated with age (OR, 0.96; 95% CI, 0.94–0.99; *p* = 0.005); tumor type; lesion size (> 5 cm: OR, 3.58; 95% CI, 1.38–9.33; *p* = 0.009); anatomical site (proximal femur: OR, 2.49; 95% CI, 1.21–5.15; *p* = 0.014; proximal humerus: OR, 3.34; 95% CI, 1.61–6.92; *p* = 0.001); and preoperative mean arterial pressure (> 110 mmHg: OR, 2.61; 95% CI, 1.20–5.67; *P* = 0.015). In subgroup analyses, after adjusting for age, tumor type, lesion size, and anatomical site, tourniquet use modified the preoperative mean arterial pressure - recurrence relationship: when tourniquet was not used, preoperative mean arterial pressure predicted local recurrence (95–110 mmHg, 4.13, 1.42–12.03, *p* = 0.009; > 110 mmHg, 28.06, 5.27–149.30, *p* < 0.001); when tourniquet was used, preoperative mean arterial pressure was not related to local recurrence (all *p* values > 0.05).

**Conclusions:**

A high preoperative blood pressure was related to local recurrence after intralesional curettage for primary bone tumors in our study. Tourniquet usage and controlling blood pressure might be beneficial for reducing local recurrence in patients scheduled to receive intralesional curettage for primary bone tumor treatment.

**Level of evidence:**

Level IV, hypothesis-generating study.

**Supplementary Information:**

The online version contains supplementary material available at 10.1186/s12891-022-05157-4.

## Introduction

Performing intralesional curettage, in lieu of *en bloc* resection, is widely accepted for the treatment of benign, intermediate, and, in some cases, low-grade malignant bone tumors [1, 2]. Patients who undergo intralesional curettage experience more rapid recovery and have better preservation of function than patients who undergo wide resection of the tumor [3]. However, tumor recurrence remains a major challenge after intralesional curettage, despite recent advances in surgical techniques and adjuvant therapies [4–6].

Generally, there are four major steps for intralesional tumor: curettage, high-speed burring, applying an adjuvant substance, and mechanical reconstruction [7]. High-speed burring can remove residual tumor tissue after curettage; thus, it has been gradually accepted and applied in musculoskeletal tumor surgery since it initially became available in the 1980s [8, 9]. Many locally applied adjuvant substances, including liquid nitrogen, phenol, ethanol, and hydrogen peroxide, have been used to extend the tumor-free margins and reduce recurrence rate [10–12].

During curettage, intramedullary hemorrhaging is commonly difficult to control when a tourniquet cannot be used, requiring blood transfusion in more than 50% of patients [13]. Hence, the extensive intramedullary hemorrhage during surgery is a potential obstacle to gain clear surgical field and complete removal of tumors [14]. GCTB and ABC usually has more blood loss during surgery and higher recurrence rate than other benign tumor [15, 16], and Zhao et al. [17] have reported massive intra-operative blood loss is an independent risk factor for local recurrence of GCTB. Based on this rationale, it is reasonable to speculate that bleeding control during surgery is crucial and high intra-operative blood pressure might increase the risk of local recurrence by increasing intramedullary hemorrhaging during surgery.

However, it was at substantial risks to maintain intra-operative blood pressure at levels much lower than preoperative baseline [18]. It is recommended to maintain intra-operative blood pressure within 80–120% of preoperative baseline values to prevent life-threatening complications [19, 20]. In clinical practice, the level of intra-operative blood pressure achieved is also largely determined by the patient’s preoperative baseline blood pressure, rather than by the willingness of the surgical team [19]. Thus, to maintain the intra-operative blood pressure at any target levels, it is necessary to evaluate and control the pre-op blood pressure baseline carefully before surgery to a level close to target [21].

We asked the following research questions: (1) Is blood pressure related to local recurrence after intralesional curettage for benign or intermediate bone tumors? (2) What’s the impact of tourniquet usage on the risk of recurrence from high blood pressure?

## Patients and methods

The study protocol was approved by and under the supervision of our institutional ethics committee (approval no. 2021–076). The procedures involved in collecting information from patients were carried out in accordance with all local laws and regulations.

### Study design, setting, and participants

**Fig. 1:**
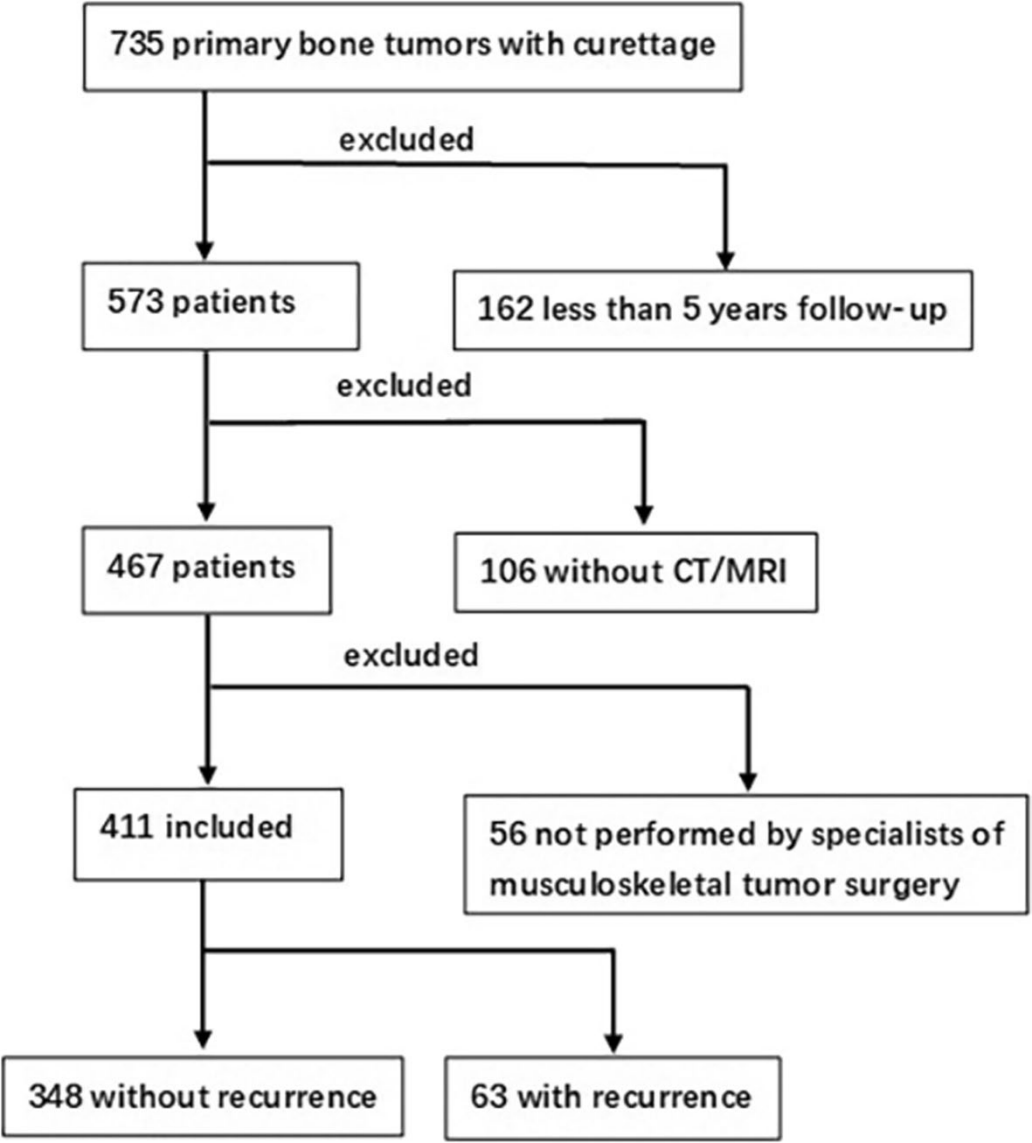
Flow chart of the study

We conducted a retrospective study of all patients with primary bone tumors treated between 2011 and 2015 with intralesional curettage at our hospital. In our institutional database, we identified 735 patients who underwent intralesional curettage for primary bone tumors during the study period. We excluded from the analysis patients who had less than 5 years of follow-up (*n* = 162); who, at baseline, lacked magnetic resonance imaging (MRI) and/or computed tomography (CT) (*n* = 106); whose surgery was not performed by specialists of musculoskeletal tumor surgery (*n* = 56). The detail flow chart of the study was shown in Fig. 1. All patients included in study did not receive preoperative denosumab treatment and preoperative embolization and no congenital abnormalities like polyostotic fibrous dysplasia were included in our study. Patient demographic and disease-related variables as well as operative details about the intralesional curettage procedure were extracted from the database.

### Baseline data collection

For focal primary bone lesions, lesion size was defined as the longest diameter of the tumor as evaluated on T1-weighted MRI, as described previously [22, 23]. Based on this maximal diameter, focal tumors were categorized into the following groups: < 2 cm, between 2 and 5 cm, or > 5 cm. If no MRI data were available, CT images were used for these calculations and groupings. When multiple focal lesions were present, lesion size was defined as the sum of the maximal diameters of all lesions.

The most frequent types of pathology were GCTB, FD, SBC/ABC, cartilaginous tumors, and chondroblastoma. Non-ossifying fibroma, lipoma, intraosseous ganglion cysts, and osteoblastomas were less frequently encountered tumor types and were categorized as “other.” A USP6 gene rearrangement test was made for diagnosis of primary ABC and secondary ABC was diagnosed by imaging and histopathology. For cases with ABC secondary to their primary tumors, classification was recorded according to their primary types. Cartilaginous tumors included atypical cartilaginous tumors (also known as grade-1 chondrosarcoma) and enchondromas. Cartilaginous tumors were diagnosed with available radiographic imaging by a multidisciplinary team (pathologists, radiologists, and orthopedic surgeons). Other tumor types were diagnosed solely by the pathologists. Anatomical localization was defined by where the proximal tumor margin was rather than by the distal one or center of tumor mass.

### Surgical treatment

All surgical procedures were performed in a similar fashion by orthopedic oncology specialists at our institution. Briefly, the lesion-bearing bone was exposed via an appropriate approach, chosen at the discretion of the treating surgeon. A large cortical window was created using a thin oscillating saw. Curettage was performed using a series of straight and angled curettes to remove all visible tumor in the bone cavity. This was followed by high-speed burring and then aggressive curettage with phenol and ethanol to expand the tumor-free margin. Finally, the cavity was filled with allografts, autografts, or polymethylmethacrylate (PMMA) for mechanical reinforcement. The use of allografts, autografts or PMMA was usually chosen by the patient in pre-surgical consultation with surgeons explaining the potential benefits and risks. The treating surgeon determined whether to perform internal fixation and which type of implant to use.

### Primary outcome measures

Postoperative follow-up was recommended at three-month intervals in the initial year and once per year after the initial year. The primary outcome was local recurrence after surgery. Local recurrence was defined by one or more of the following being present: (1) diagnostic histology of suspected tumor tissues obtained from biopsy or re-operation, (2) a static or slow-growing lesion with typical radiological finding(s) of local recurrence when re-operation is not indicated. The anesthesiologist measured intra-operative hemorrhage volume, which was determined by summing the intra-operative suction fluid volume and the volume accumulated from surgical gauzes, according to the gravimetric method [24]. The preoperative mean arterial pressure (pre-op MAP) was cuff-measured defined as follows [25]: $pre- op\ MAP=\frac{2\times \mathrm{diastolic}\ \mathrm{blood}\ \mathrm{pressure}+\mathrm{systolic}\ \mathrm{blood}\ \mathrm{pressure}}{3.}$  

### Statistical analyses

Continuous variables were summarized as means ± standard deviation (SD); categorical variables were summarized as counts with percentages (%), unless otherwise noted. All statistical group comparisons were two-sided and were considered significant at *p* < 0.05, unless otherwise noted. IBM SPSS version 26.0 (IBM Corp. Released 2019. IBM SPSS Statistics for Windows, Version 26.0; Armonk, NY: IBM Corp.) was used. Univariable comparisons were evaluated with Fisher’s exact test or the Mann-Whitney U test, depending on whether the variables were categorical or continuous, respectively. When a yielded *p* value in the univariable analysis was *p* < 0.2, this variable was assessed in multivariable analysis to identify independent risk factors. Comparisons of local recurrence between patients grouped by tourniquet usage (use/non-use) were adjusted for patient- and disease-related variables from the multivariable analysis. Other variables were evaluated using Dunnett’s test for multiple comparisons.

## Results

### General information

Data from 411 patients were available for analysis (Table 1). At an average follow-up of 6.8 ± 1.0 years (5.0–9.4 years), local recurrence was found in 63 of 411 patients (15.3%) of this study. Ninety-six patients had GCTBs, 102 had FDs, 71 had SBC/ABCs, 88 had cartilaginous tumors, 30 had chondroblastomas, and 24 had other types of tumors. The distal femur and more distal locations (*n* = 163) were the most frequent site of tumors, followed by the distal humerus and more distal locations (*n* = 95), proximal femur (*n* = 69), proximal humerus (*n* = 59), and pelvis (*n* = 25).

**Table 1:** Results of univariable analysis of patient-, surgical-, and disease-related characteristics in predicting local recurrence of bone tumor after intralesional curettage

Variable	No local recurrence (*n* = 348)	Local recurrence (*n* = 63)	***p*** value*
Mean age (y) at baseline, mean ± SD	37.1 ± 9.8	33.4 ± 11.1	0.002
Follow up duration (y), mean ± SD	6.8 ± 1.0	6.8 ± 0.9	0.811
Sex, n (%)			
Male	194 (55.7)	37 (58.7)	0.681
Female	154 (44.3)	26 (41.3)	
Lesion size, n (%)			
< 2 cm	43 (12.3)	5 (7.9)	0.002
2–5 cm	168 (48.3)	18 (28.6)	
> 5 cm	137 (39.4)	40 (63.5)	
Tumor type, n (%)			
Giant cell tumor of bone (GCTB)	73 (21.0)	23 (36.5)	0.032
Fibrous dysplasia (FD)	84 (24.1)	18 (28.6)	
Simple/Aneurysmal bone cyst (SBC/ABC)	62 (17.8)	9 (14.3)	
Cartilaginous tumor	82 (23.6)	6 (9.5)	
Chondroblastoma	25 (7.2)	5 (7.9)	
Other	22 (6.3)	2 (3.2)	
Preoperative mean arterial pressure (pre-op MAP), n (%)			
< 95 mmHg	178 (51.1)	25 (39.7)	0.065
95–110 mmHg	143 (41.1)	28 (44.4)	
> 110 mmHg	27 (7.8)	10 (15.9)	
Pathologic fracture, n (%)	40 (11.5)	6 (9.5)	0.828
Internal fixation, n (%)	253 (72.7)	50 (79.4)	0.350
Anatomical site, n (%)			
Distal femur and more distal locations	145 (41.7)	18 (28.6)	0.027
Proximal femur	55 (15.8)	14 (22.2)	
Distal humerus and more distal locations	84 (24.1)	11 (17.5)	
Proximal humerus	43 (12.4)	16 (25.4)	
Pelvis, scapula and clavicle	21 (6.0)	4 (6.3)	
Previous recurrence, n (%)	19 (5.5)	5 (7.9)	0.392
Reconstructive method, n (%)			
Polymethylmethacrylate (PMMA)	38 (10.9)	9 (14.3)	0.890
Allograft	217 (62.4)	38 (60.3)	
Autograft	56 (16.1)	10 (15.9)	
Mixed graft (autograft + allograft)	37 (10.6)	6 (9.5)	
ABC, n (%)			
Presence	61 (17.5)	14 (22.2)	0.375

*Mann-Whitney tests or Fisher’s exact tests were used, depending on whether the variables were continuous or categorical

### Patient demographic and disease-related factors predict local recurrence after Intralesional curettage

**Fig. 2:**
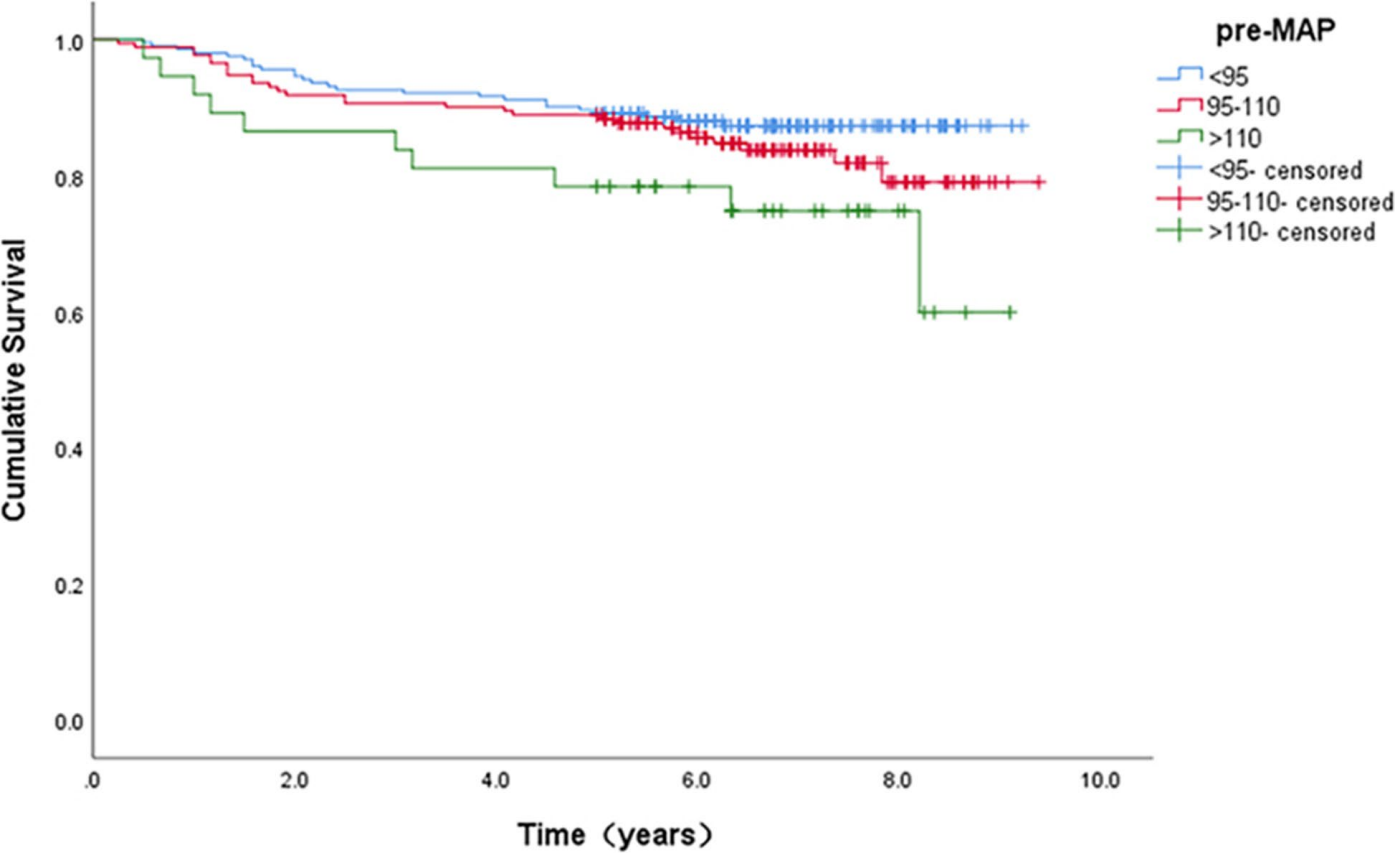
Result of Kaplan-Meier survival analysis among three different blood pressure groups

Table 1 summarizes and compares demographic, clinical, and disease-related characteristics of the 411 bone-tumor patients grouped by presence or absence of local recurrence. The overall local recurrence rate was 15.3% (63/411) within an average surgery-recurrence interval of 3.0 ± 2.1 years (range, 0.3–8.2 years). The average age in local recurrence group was significantly younger than that in the non-local recurrence group (33.4 versus 37.1 years, *p* = 0.002). Fibrous dysplasia (FD) and giant cell tumor of bone (GCTB) accounted for the largest proportion (24.8 and 23.4%, respectively), and GCTB had the highest local recurrence rate (24.0%). An increasing trend in local recurrence rate was observed with ascending of the blood pressure interval (< 95 mmHg, 12.3% vs. 95–110 mmHg, 16.4% vs. > 110 mmHg, 27.0%; *p* = 0.065). The Kaplan-Meier survival curve (Fig. 2) showed absolute but statistically insignificant worse prognosis (*p* = 0.065) for recurrence-free survival in patients with high pre-op MAP (> 110 mmHg). Age, tumor type, lesion size, anatomical site, and pre-op MAP yielded *p* values of < 0.2 and thus were entered as independent variables in the multivariable analyses.

In the multivariable Cox analysis (Table 2), local recurrence was associated with age (OR, 0.96; 95% CI, 0.94–0.99; *p* = 0.005); tumor type; lesion size (> 5 cm: OR, 3.58; 95% CI, 1.38–9.33; *p* = 0.009); anatomical site (proximal femur: OR, 2.49; 95% CI, 1.21–5.15; *p* = 0.014; proximal humerus: OR, 3.34; 95% CI, 1.61–6.92; *p* = 0.001); and pre-op MAP (> 110 mmHg: OR, 2.61; 95% CI, 1.20–5.67; *P* = 0.015). The results of single disease analysis (GCTB, Enchondroma/ Atypical cartilaginous tumor, and Fibrous Dysplasia) were showed in the Supplementary material (Tables S1-S5).

**Table 2:** Results of multivariable Cox regression to identify significant prognostic factors

Variable	Adjusted OR	95% CI	***p*** value
Age	0.96	0.94–0.99	0.005
Lesion size			
< 2 cm	Reference^a^		
2–5 cm	1.57	0.57–4.34	0.383
> 5 cm	3.58	1.38–9.33	0.009
Tumor type			
Giant cell tumor of bone (GCTB)	Reference^a^		
Fibrous dysplasia (FD)	0.46	0.24–0.88	0.019
Cartilaginous tumors	0.18	0.07–0.46	< 0.001
Simple/Aneurysmal bone cyst (SBC/ABC)	0.35	0.15–0.78	0.010
Chondroblastoma	0.50	0.18–1.37	0.179
Other	0.18	0.04–0.80	0.024
Anatomical site			
Distal femur and more distal locations	Reference^a^		
Proximal femur	2.49	1.21–5.15	0.014
Distal humerus and more distal locations	1.75	0.80–3.83	0.159
Proximal humerus	3.34	1.61–6.92	0.001
Pelvis, scapula and clavicle	2.48	0.81–7.62	0.112
Preoperative mean arterial pressure (pre-op MAP)			
< 90 mmHg	Reference^a^		
90–105 mmHg	1.51	0.86–2.62	0.149
> 105 mmHg	2.61	1.20–5.67	0.015

^a^Reference category was chosen using the lowest β coefficient

*OR* Odds ratio, *CI* Confidence interval

### Does tourniquet usage modify the risk of local recurrence in patients with high pre-op MAP?

After we stratified patients by intra-operative tourniquet usage (use/non-use), we found that the pre-op MAP–recurrence relationship was modified by tourniquet usage (Table 3). For patients who had a tourniquet applied during intralesional curettage (*n* = 255), and when age, tumor type, lesion size, and anatomical site variables were statistically controlled for, pre-op MAP (95–110 mmHg, > 110 mmHg, both *p*’s > 0.5; Table 3) was not related to local recurrence. However, for patients who did not have a tourniquet applied (*n* = 156), local recurrence risk was significantly related to pre-op MAP (95–110 mmHg: OR, 4.13; 95% CI, 1.42–12.03; *p* = 0.009; > 110 mmHg: OR, 28.06; 95% CI, 5.27–149.30; *p* < 0.001; Table 3).

**Table 3:** Comparison of outcomes in patients stratified by pre-op MAP and tourniquet use/non-use^a^

Variable	Pre-op MAP categories			
	95–110 mmHg		> 110 mmHg	
	Mean differences (95% CI)	***p*** value	Mean differences (95% CI)	***p*** value
**Overall (*n*** **= 411)**				
Local recurrence ^b^	1.59 (0.84–3.00)	0.150	3.28 (1.26–8.52)	0.015
Intra-operative hemorrhage volume (mL)	89.9 (−15.5–195.2)	0.108	232.3 (50.9–413.7)	0.009
Duration of surgery (min)	5.0 (−1.2–11.2)	0.137	8.6 (−2.1–19.2)	0.139
Intra-operative hemorrhage velocity (mL/min)	0.47 (−0.52–1.46)	0.490	1.39 (−0.31–3.09)	0.130
**Tourniquet use (*n*** **= 255)**				
Local recurrence ^b^	0.77 (0.32–1.86)	0.564	0.59 (0.07–5.07)	0.629
Intra-operative hemorrhage volume (mL)	6.7 (−5.2–18.7)	0.366	3.1 (−17.6–23.8)	0.928
Duration of surgery (min)	0.1 (−7.2–7.3)	1.00	3.8 (−8.7–16.2)	0.742
Intra-operative hemorrhage velocity (mL/min)	0.16 (−0.05–0.37)	0.178	0.01 (−0.37–0.36)	0.999
**Tourniquet non-use (*n*** **= 156)**				
Local recurrence ^b^	4.13 (1.42–12.03)	0.009	28.06 (5.27–149.30)	< 0.001
Intra-operative hemorrhage vol (mL)	271.8 (188.8–354.8)	< 0.001	628.9 (486.4–771.5)	< 0.001
Duration of surgery (min)	14.3 (5.2–23.3)	0.001	16.9 (1.3–32.5)	0.031
Intra-operative hemorrhage velocity (mL/min)	1.38 (0.27–2.50)	0.011	3.88 (1.97–5.80)	< 0.001

^a^Intra-operative hemorrhage volume, surgical duration, and intra-operative hemorrhage velocity are presented as mean differences (95% CI) compared to the group having < 95 mmHg pre-op MAP

^b^Local recurrence was presented as adjusted OR (95% CI) with reference to the group having < 95 mmHg pre-op MAP

Intra-operative hemorrhage velocity = intra-operative hemorrhage volume/surgical duration; pre-op *MAP* Preoperative mean arterial pressure; *OR* Odds ratio, *CI* Confidence interval

There was evidence in support of our hypothesis that in patients with high pre-op MAP who underwent intralesional curettage without a tourniquet, intra-operative hemorrhage volume and hemorrhage velocity were higher (Table 3). By contrast, with tourniquet usage during intralesional curettage, the mean intra-operative hemorrhage volume and velocity were not different. Possibly as a result of interference from increased hemorrhaging, the surgical duration was also longer when a tourniquet was not used (Table 3).

## Discussion

Intralesional curettage is a widely used treatment for primary benign bone tumors, while local recurrence of tumors remains a major challenge after curettage [2, 6]. Previous studies have reported massive intra-operative blood loss is an independent risk factor for local recurrence [15, 17], and we mainly focused on assessing the risk related to patients’ blood pressure instead of hemorrhage in the present study, because measuring blood pressure could provide a unified assessment approach in case of different tumor types and anatomical sites. Finally, we found high preoperative blood pressure was related to the risk of local recurrence after intralesional curettage for primary bone tumors, and tourniquet usage might be beneficial for reducing the risk of recurrence from high blood pressure.

Many previous studies have suggested patients might be at increased risk of local recurrence when the tumor was localized at more proximal sites (i.e. proximal femur and humerus) [26–31]. As a possible explanation, because tourniquet use is impossible for tumors localized in the proximal humerus or femur, intramedullary hemorrhage during surgery is generally uncontrollable during curettage, especially for highly vascularized tumors. Thus, extensive intramedullary hemorrhaging might hinder thorough tumor removal, leading to an increased local recurrence rate [14, 17]. Besides, uncontrolled bleeding might blunt the effects of adjuvant agents and it constitutes a continuous heat exchange mechanism could neutralize the thermal effects of cryosurgery, and a dilutional effect could neutralize the chemical effects of topical agents like ethanol and phenol [7, 32]. With this rationale, in the absence of tourniquet control, high intra-operative blood pressure could increase intramedullary hemorrhaging, obscuring the surgical field, leading to incomplete tumor removal and increased risk of local recurrence. Besides, the level of intra-operative blood pressure achieved is also largely determined by the patient’s preoperative baseline blood pressure in clinical practice [19]. In the present study, we mainly focused on assessing the risk related to patients’ pre-op MAP instead of intra-operative blood pressure and hemorrhage, which were clearly more directly relevant. The results of statistical analysis showed that a pre-op MAP > 95 mmHg was related to local recurrence, and intra-operative blood pressure was an independent risk factor when tourniquet usage is precluded. Controlling blood pressure to a reasonably low level might be beneficial for reducing local recurrence in patients scheduled to receive intralesional curettage for primary bone tumor treatment. Moreover, although it’s easy to control the blood pressure of young patients, there would exist potential risks if intra-operative blood pressure was arbitrarily determined by anesthetists during surgery, and it is still recommended to maintain intra-operative blood pressure within 80–120% of preoperative baseline values [19, 20].

Tourniquet control, if used during intralesional curettage, could create a hemorrhage-free surgical field and thus could potentially modify the risk from high blood pressure. Thus, we further investigated the risk of blood pressure in subgroups of patients according to tourniquet use/non-use and found that the risk of intra-operative blood pressure was derived from cases lacking tourniquet use. It is preferable to maintain intra-operative blood pressure within 20% of preoperative baseline values [18]. Thus, relationship between preoperative blood pressure and postoperative mortality is described by a “J-shaped” curve, in which risk is the lowest when MAP is approximately 95 mmHg [19]. Thus, based on this outcome, controlling blood pressure to a reasonably low level might be beneficial for patients scheduled to receive intralesional curettage for a primary bone tumor in that it could appreciably reduce local recurrence rate. Achieving reasonable blood pressure control might require multidisciplinary interventions, involving surgeons, cardiologists, and anesthesiologists.

Another risk factor for local recurrence in our study was being younger at the time of surgery for intralesional curettage. This finding is consistent with the hypothesis that tumor cells tend to be more active and aggressive in younger patients [33, 34]. Another possible explanation for this phenomenon is that patients with more active and aggressive tumors were more likely to be symptomatic and thus diagnosed at a young age [35, 36]. This issue remains equivocal, and future investigations are needed to make definitive conclusions.

### Limitation

This study was a retrospective evaluation and had several limitations imposed by such a design. First, due to its retrospective design, the possibility of selection bias might be present. However, all patients receiving intralesional curettage for their primary bone tumor during the study period were reviewed and rigorously assessed for eligibility, which should minimize the impact of selection bias. Second, phenol and ethanol for aggressive curettage were the preferred adjuvant treatment in the study period, while other adjuvant such as liquid nitrogen or argon gas for cryoablation was not available in our hospital, and its thermal effect might be neutralized by a continuous heat exchange mechanism of uncontrolled bleeding [32]. There is no consensus on optimal adjuvant agents, and the margin-expanding capabilities of these agents are generally similar to other options [7]. Thus, this choice of adjuvant agents in our study might or might not affect the main conclusions.

Although primary or intermediate, inter-tumoral heterogeneity between different tumors inevitably existed for analysis of such different bone tumors. We did not find significant difference among groups of pre-op MAP in the single disease analysis but we could observe an absolute difference in local recurrence rate (e.g. GCTB; < 95 mmHg, 21.6% vs. 95–110 mmHg, 22.9% vs. > 110 mmHg, 40.0%; *p* = 0.45), similar results could also be found in non-vascularized lesions (Table S1-S3). In multivariable Cox regression analysis of GCTB, we observed a large absolute but statistically insignificant odds ratio in recurrence rate between groups of pre-op MAP < 95 mmHg and > 110 mmHg (Table S4). The result was reasonable because the risk factor of local recurrence for GCTB was intricate. Previous studies have found age [37–39], Campanacci classification [40, 41], tumor sites [31, 42, 43], inflammation related factors [44, 45], and image related factors [46–48] were potential risk factors of recurrence. Due to the exploratory nature of this study, the sample size of single tumor was relatively small and it was lack of including other more detailed and potential confounding factors, which had a certain impact on the analysis results. Thus, we reviewed a large patient population of multiple disease over a relatively short time interval length of follow up (2011–2015), and analyzed our data after stratification to reduce potential bias.

Finally, as a retrospective study, intra-operative blood pressure was not available in this research and we focused on patients’ pre-op MAP instead, hence the findings in this study might not be so conclusive and further larger prospective study should be needed for validation.

## Conclusions

In conclusion, a high blood pressure was related to local recurrence after intralesional curettage for primary bone tumors in our study. Tourniquet usage and controlling blood pressure to a reasonably low level might be beneficial for reducing local recurrence in patients scheduled to receive intralesional curettage for primary bone tumor treatment.

## Supplementary Information

**Additional file 1.**

## Data Availability

The datasets used and/or analysed during the current study are available from the corresponding author on reasonable request.

## Abbreviations

pre-op MAP: Preoperative mean arterial pressure
OR: Odds ratio
MRI: Magnetic resonance imaging
CT: Computed tomography
GCTB: Giant cell tumor of bone
FD: Fibrous dysplasia
SBC/ABC: Simple/Aneurysmal bone cyst
PMMA: Polymethylmethacrylate
SD: Standard deviation
CI: Confidence interval
