# Supplementary material for: The blood pressure and use of tourniquet are related to local recurrence after intralesional curettage of primary benign bone tumors: a retrospective and hypothesis-generating study
Source: BMC Musculoskelet Disord. 2022 Mar 3;23:201. doi: 10.1186/s12891-022-05157-4 (PMC8892695; doi:10.1186/s12891-022-05157-4)
Supplement: Supplementary file 1 — Additional file 1. [file 12891_2022_5157_MOESM1_ESM.docx]

**Table S1**. Results of univariable analysis of patients with GCTB

| Preoperative mean arterial pressure (pre-op MAP), n (%) | **No local recurrence**  (n=73) | **Local recurrence** (n=23) | **p value** |
| --- | --- | --- | --- |
| <95 mmHg | 40 (78.4) | 11 (21.6) | 0.45 |
| 95-110 mmHg | 27 (77.1) | 8 (22.9) |  |
| >110 mmHg | 6 (60.0) | 4 (40.0) |  |

**Table S2**. Results of univariable analysis of patients with Enchondroma/ Atypical cartilaginous tumor

| Preoperative mean arterial pressure (pre-op MAP), n (%) | **No local recurrence**  (n=82) | **Local recurrence** (n=6) | **p value** |
| --- | --- | --- | --- |
| <95 mmHg | 42 (100.0) | 0 (0.0) | n.a. |
| 95-110 mmHg | 32 (88.9) | 4 (11.1) |  |
| >110 mmHg | 8 (80.0) | 2 (20.0) |  |

**Table S3.** Results of univariable analysis of patients with Fibrous Dysplasia

| Preoperative mean arterial pressure (pre-op MAP), n (%) | **No local recurrence**  (n=84) | **Local recurrence** (n=18) | **p value** |
| --- | --- | --- | --- |
| <95 mmHg | 42 (87.5) | 6 (12.5) | 0.44 |
| 95-110 mmHg | 32 (78.0) | 9 (22.0) |  |
| >110 mmHg | 10 (76.9) | 3 (23.1) |  |

**Table S4.** Results of pre-op MAP in multivariable Cox regression analysis of GCTB

| Preoperative mean arterial pressure (pre-op MAP) | **Adjusted OR** | **95% CI** | **p value** |
| --- | --- | --- | --- |
| <90 mmHg | Reference* | | |
| 90-105 mmHg | 0.99 | 0.39-2.51 | 0.973 |
| >105 mmHg | 3.53 | 0.70-17.81 | 0.127 |

**Table S5.** Results of pre-op MAP in multivariable Cox regression analysis of Fibrous Dysplasia

| Preoperative mean arterial pressure (pre-op MAP) | **Adjusted OR** | **95% CI** | **p value** |
| --- | --- | --- | --- |
| <90 mmHg | Reference* | | |
| 90-105 mmHg | 1.35 | 0.45-3.99 | 0.592 |
| >105 mmHg | 1.67 | 0.39-7.25 | 0.492 |
